# The neuroscience of social conformity: implications for fundamental and applied research

**DOI:** 10.3389/fnins.2015.00337

**Published:** 2015-09-28

**Authors:** Mirre Stallen, Alan G. Sanfey

**Affiliations:** ^1^Department of Psychology, Stanford UniversityStanford, CA, USA; ^2^Behavioural Science Institute, Radboud University NijmegenNijmegen, Netherlands; ^3^Donders Institute for Brain, Cognition and BehaviourNijmegen, Netherlands

**Keywords:** social conformity, decision making, policy implications, functional magnetic resonance imaging, behavioral change

## Abstract

The development of closer ties between researchers and practitioners in the domain of behavior and behavioral change offers useful opportunities for better informing public policy campaigns via a deeper understanding of the psychological processes that operate in real-world decision-making. Here, we focus on the domain of social conformity, and suggest that the recent emergence of laboratory work using neuroscientific techniques to probe the brain basis of social influence can prove a useful source of data to better inform models of conformity. In particular, we argue that this work can have an important role to play in better understanding the specific mechanisms at work in social conformity, in both validating and extending current psychological theories of this process, and in assessing how behavioral change can take place as a result of exposure to the judgments of others. We conclude by outlining some promising future directions in this domain, and indicating how this research could potentially be usefully applied to policy issues.

## Introduction

Recent innovative work in applied psychology has established that making people aware of the behavior of others is a useful technique for inducing positive behavioral change on a societal level. For example, taxpayers are more likely to pay what they owe when knowing that others do (Coleman, 2007; Cabinet Office UK Behavioural Insights Team, 2012), householders decrease their energy use when informed that they use more power than their neighbors (Schultz et al., 2007; Slemrod and Allcott, 2011), and people are more likely to give to a charity if it is viewed as the social norm (Alpizar et al., 2008; Smith et al., 2015). Many of these strategies have been successfully applied in recent years, albeit on a somewhat *ad hoc* basis. However, a better understanding of the mechanisms of **social influence** and **conformity**, both cognitively and neurally, is important in extending these techniques into other domains of interest to policy-makers.

Over the course of the last decade, a growing body of work has examined the neurocognitive correlates of social influence (for reviews see Falk et al., 2012; Morgan and Laland, 2012; Izuma, 2013; Schnuerch and Gibbons, 2014; Cascio et al., 2015). These studies have focused on diverse aspects of social influence, ranging from how the opinion of others affects the valuation and perception of simple stimuli (Berns et al., 2005; Mason et al., 2009; Chen et al., 2012; Stallen et al., 2013; Tomlin et al., 2013; Trautmann-Lengsfeld and Herrmann, 2013) to more complex, realistic, choice options (Klucharev et al., 2009; Berns et al., 2010; Campbell-Meiklejohn et al., 2010; Zaki et al., 2011; Huber et al., 2015), and finally, to what brain mechanisms underlie long-term conformity, how the mere presence of peers impacts brain activity and leads to changes in risk-taking and trust decisions (Steinberg, 2007; Chein et al., 2011; Fareri et al., 2012, 2015), and how the brain reconciles misleading influence (Edelson et al., 2011, 2014; Izuma, 2013). The goal of this Focused Review is not to re-summarize this work, but rather to explore to what extent these neuroimaging studies can contribute to our understanding of the psychology of social influence, and what promising directions lie ahead in the future. Specifically, while social influence is a broad term describing the impact of others on our behavior and opinions, we here focus on studies on conformity, with conformity referring to the actual alignment of people's opinions or behaviors with those of others. This review is structured around three ways in which neuroimaging has been suggested to contribute to psychology (Moran and Zaki, 2013), namely the role of neuroimaging in (i) identifying the fundamental mechanisms that underlie behavior, (ii) dissociating between psychological theories that make similar behavioral predictions, and (iii) using brain activity to predict subsequent behavioral change.

KEY CONCEPT 1 **Social influence**

The influence of others on our attitudes, opinions, and behaviors. Social influence can take many forms, including conformity (see Key concept 2), reactance (deliberately adopting a view contrary to that of others), persuasion (changing one's view based on appeals to reason or emotion), and minority influence (when an individual or small group exerts influence on the majority).

KEY CONCEPT 2 **Conformity**

Aligning one's attitude, opinion or behavior to those of others. Social psychology distinguishes between two reasons for conformity. Informational conformity occurs when one adopts the view of others because others are assumed to possess more knowledge about the situation. Normative conformity refers to the act of conforming to the positive expectations of others in order to be liked and accepted by them.

## Mechanisms of conformity

A growing number of neuroscientific studies suggest that conformity recruits neural signals that are similar to those involved in **reinforcement learning** (Klucharev et al., 2009; Campbell-Meiklejohn et al., 2010; Kim et al., 2012; Shestakova et al., 2013). For example, in the study by Klucharev et al. (2009), participants were asked to rate female faces and then saw the purported aggregate judgments of other raters. Upon seeing those faces a second time, participants' ratings were shown to shift in the direction of the group judgments. Neuroimaging results demonstrated that when individual ratings differed from those of the group, activity in the rostral cingulate zone, an area in the medial prefrontal cortex and involved in the processing of conflict (Ridderinkhof et al., 2004), increased, while activity in the nucleus accumbens, an area associated with the expectation of reward (Knutson et al., 2005), decreased. Interestingly, the amplitude of these signals predicted conformity, such that when this incongruence was large (although exactly what magnitude this discrepancy should be to trigger conformity is still undetermined), people then adjusted their behavior and aligned their opinion with that of the group (Klucharev et al., 2009). Similar neural discrepancy signals reflecting the deviation of one's own assessment and a salient external opinion have been reported by other studies as well (Campbell-Meiklejohn et al., 2010; Deuker et al., 2013; Izuma and Adolphs, 2013; Lohrenz et al., 2013).

KEY CONCEPT 3 **Reinforcement learning**

Reinforcement learning is learning about the environment by trial and error. By encountering positive and negative outcomes, individuals learn over time what action to select to maximize reward. In conformity research, acceptance by the group is typically seen as the reward and matching one's attitude, opinion or behavior with those of others as the means to achieve this outcome.

Consistent with previous work showing that regions in the medial prefrontal cortex are associated with behavioral adjustment following both positive/negative or unexpected outcomes (Ridderinkhof et al., 2004), activity in this region, slightly more anterior than the medial frontal activity reported by Klucharev et al. (2009), has been found to encode not only conformity toward the liked group, but has also been shown to correlate with behavioral adjustments away from the disliked group (Izuma and Adolphs, 2013, and see Izuma, 2013 for an overview of medial frontal activations in social conformity studies). To test the causal role of the medial frontal cortex in conformity, researchers used transcranial magnetic stimulation (TMS) to temporarily down-regulate this area in order to examine whether this interfered with behavioral adjustments to group opinions (Klucharev et al., 2011). Indeed, transient down-regulation of this region appeared to reduce behavioral change, confirming the critical involvement of the posterior medial prefrontal cortex in conformity. We believe that this research demonstrates a clear role for functional neuroimaging in better elucidating the precise systems that underpin social conformity. While we have used the mechanism of reinforcement learning here as an example of how we can better understand complex social behavior by examining basic processes, future investigations are required to gain more insight into the exact processes underlying conformity. For instance, it is unknown to date whether deviation from the group opinion triggers actual dopamine-dependent reward prediction error signals, or whether conformity is processed in different ways.

## Validating psychological theories

In addition to identifying more precisely the neural mechanisms of conformity, neuroscience can help to adjudicate between competing psychological theories that make similar behavioral predictions with regard to the reason why people conform. For instance, one of the first neuroimaging studies on social influence aimed to ascertain whether conformity is a function of an explicit decision to match the choices of others, or whether the presence of others actually changes individuals' true perception or attentional focus (Berns et al., 2005). By using fMRI and a mental rotation task, the authors examined the neural correlates of conformity in the face of incorrect peer feedback regarding the degree of rotation of an abstract figure. Conforming to incorrect feedback altered activity within visual cortical and parietal regions that were involved in performance of the mental rotation task itself. Based on the involvement of these regions in perception and based on the absence of activity in frontal decision-making regions the authors concluded that behavioral change in this study was due to a modification of low-level perceptual processes as opposed to a decision to conform taken at an executive level. Though caution is warranted when using these types of reverse inference techniques to establish knowledge of precise cognitive processes (Poldrack, 2006), additional support for the hypothesis that social conformity can affect basic cognitive processing comes from electroencephalography (EEG) work showing that deviation from the norm of a peer group can impact early visual brain signals (Trautmann-Lengsfeld and Herrmann, 2013, 2014).

Another focus of neuroimaging research has been to investigate whether viewing the opinion of others can actually change individuals' true preferences, testing social psychological theories which distinguish genuine attitude modifications from mere public **compliance** in which people conform without changing their true attitude (Cialdini and Goldstein, 2004). This direction has shown promise, demonstrating that social influence moderates activity in the striatum and ventromedial prefrontal cortex. These two brain areas are known to be involved in the processing of rewards, and are believed to work in concert to encode subjective value (Bartra et al., 2013). Signal across these areas was enhanced when participants viewed simple, abstract symbols that had been rated in popularity by peers (Mason et al., 2009), in addition to when participants were presented with actual concrete stimuli such as faces and songs that were liked by others (Klucharev et al., 2009; Campbell-Meiklejohn et al., 2010; Zaki et al., 2011). Together, these findings suggest that the behavior and opinion of others can in fact directly impact the neural representation of value associated with particular stimuli, and demonstrate how neuroimaging can help in disentangling true conformity from simple public compliance. As such, this approach provides valuable information in validating and extending psychological theories of conformity.

KEY CONCEPT 4 **Compliance**

Compliance refers to a superficial form of conformity when individuals express the same opinion or behavior as the group but do not change their actual underlying attitude or belief. Compliance is also known as public conformity and is the opposite of private conformity, or internalization, when people truly believe the group is right and actual preference change occurs.

## Predicting behavioral change

A third way by which neuroscience research may contribute to a better understanding of social influence is in its ability to use brain data to directly predict behavior. For example, the strength of the discrepancy signal in response to a conflict between one's own judgment and that of a group not only predicted subsequent conformity, but activity within the striatum also correlated with individual differences, with participants who adjusted their opinion in response to group disagreement showing lower activations in this area than participants who did not adjust their views (Klucharev et al., 2009). Individual differences in the tendency to align one's behavior with the group have also been associated with functional and structural differences in the orbitofrontal cortex (Campbell-Meiklejohn et al., 2012a; Charpentier et al., 2014). Additionally, these tendencies can be modulated by administration of oxytocin (Stallen et al., 2012), a hormone involved in a wide range of social behaviors, as well as methylphenidate, an indirect dopamine and noradrenalin agonist (Campbell-Meiklejohn et al., 2012b).

An interesting extension to this laboratory research, and one that received relatively little attention to date, is to what extent neural activity can predict actual long-term behavioral change, as measured in real-world decisions. One study showed that the discrepancy signal in the medial frontal cortex could predict preference change several months later (Izuma and Adolphs, 2013). However, this finding could potentially be explained by the general tendency to be consistent with one's own previous behavior, since participants had already explicitly rated the stimuli once before in this experiment. A follow-up study that circumvented this issue demonstrated robust conformity effects whereby judgments of facial attractiveness were altered by knowing the opinions of others, with this effect lasting up to 3 days (Huang et al., 2014). Persistent conformity effects were also found in a study examining the impact of social pressure on memory change (Edelson et al., 2011). Participants in this study were exposed to incorrect recollections of other co-observers while being asked questions about a documentary they had viewed. After a week's delay they were tested again, and though they were informed that the answers they had heard before were actually determined randomly, participants nonetheless still showed a strong tendency to conform to the erroneous recollections of the group, with, importantly, neuroimaging data indicating that social influence modified the neural representation of the memories. Specifically, both activity in the amygdala at the time of exposure to social influence, as well as the strength of connectivity between this area and the hippocampus, predicted long-lasting, persistent memory errors. Future progress in this field could usefully focus on how this work extends to the public health arena, as discussed in the following section.

## Conclusion and future directions

Though in its relative infancy in terms of a substantive body of experimental research, neuroscience, and in particular functional neuroimaging, has a great deal to offer the study of social influence. Knowledge of the neural mechanisms underlying conformity can be used to constrain existing psychological theories, as well as to construct novel ones, and can help in understanding what precise cognitive processes are engaged. To achieve this, a productive next step is to better understand how to interpret brain activity. For instance, does the discrepancy signal in the medial frontal cortex in response to a conflict between one's own opinion and that of a group reflect the process of cognitive reappraisal and subsequent attitude adjustment, or rather does it indicate an increase in negative affect which in turn can motivate behavioral change? Other interpretations are also possible, for example theories that medial frontal activity reflects recruitment of theory of mind processes (Gallagher and Frith, 2003), the experience of conflict (Pochon et al., 2002; Klucharev et al., 2009), or, more generally, a violation of expectations (Chang and Sanfey, 2013). Of course, brain areas are typically not selectively engaged in a single psychological process but rather are implicated in multiple computations, and therefore the interpretation of brain activity based solely on the findings from the research outlined here is challenging. Naturally, the increasing number of studies in this area will help in delineating the precise processes involved, and converging methodological approaches also have promise in this regard. For example, additional data from independent localizer tasks within the same participants can be helpful in determining the psychological process in which a brain area is engaged (Zaki et al., 2011; Izuma and Adolphs, 2013), and the use of meta-analyses, functional connectivity approaches assessing neural network computations, and large-scale databases can also help reduce the potential pool of hypotheses (Poldrack, 2011). One useful online meta-analysis database is the platform Neurosynth, which allows for large-scale automated meta-analyses of functional magnetic resonance imaging (fMRI) data (Yarkoni et al., 2011).

We suggest that one specific promising future direction for neuroscience to contribute to the understanding of social influence is to further investigate the emotions that drive behavioral adjustments due to conformity. For instance, people may align their preferences with others because they affiliate and thereby feel a need to belong to a group (Tafarodi et al., 2002; Cialdini and Goldstein, 2004). However, negative emotions, such as the fear of social exclusion or a sense of shame or guilt in having differing opinions, could also be drivers of conformity (Janes and Olson, 2000; Berns et al., 2010; Yu and Sun, 2013). Combining neuroscientific methodologies with clever behavioral paradigms can provide substantially greater insight into the specific emotions that underlie conformity in a given context, as accumulating evidence suggests that neuroimaging data can support inferences about affective states (Knutson et al., 2014). The use of innovative methods, including multivariate brain imaging techniques, can be expected to improve the mapping of brain activity onto both affective experience and behavior in the near future (Formisano and Kriegeskorte, 2012).

The accumulating laboratory evidence allied with these aforementioned likely future developments demonstrates great promise in constructing improved neural and psychological models of social conformity. A better understanding of the processes that drive conformity is not only interesting from a scientific perspective, but also provides relevant practical insights for social policy. Policy campaigns often attempt to motivate behavioral change by the use of social influence, such as programs discouraging smoking among adolescents by emphasizing peer disapproval, or reducing alcohol consumption at schools by correcting prevalent, though false, beliefs about the behavior of others (Neighbors et al., 2004; Youth smoking prevention: truth campaign USA^1^). Although social influence campaigns such as these can sometimes be effective, there are also many cases in which they fail (Clapp et al., 2003; Granfield, 2005). Deeper understanding of the processes that both facilitate and prevent social conformity will undoubtedly help to predict when, and how, behavioral change can occur, and has the potential to provide useful hypotheses that can be tested in real-world field experiments.

### Conflict of interest statement

The authors declare that the research was conducted in the absence of any commercial or financial relationships that could be construed as a potential conflict of interest.

## Footnotes

^1^http://www.legacyforhealth.org
